# Repetitive Transcranial Magnetic Stimulation in Major Depressive Disorder: From Bench to Bedside—A Scoping Review of Neurobiological Mechanisms and Clinical Translation

**DOI:** 10.3390/bioengineering13030288

**Published:** 2026-02-28

**Authors:** Deborah Maria Trandafir, Florin Zamfirache, Cristina Dumitru, Beatrice Mihaela Radu, Adela Magdalena Ciobanu

**Affiliations:** 1 Neuroscience Department, Discipline of Psychiatry, Faculty of Medicine, “Carol Davila” University of Medicine and Pharmacy, 020021 Bucharest, Romania; deborah-maria@drd.umfcd.ro (D.M.T.); adela.ciobanu@umfcd.ro (A.M.C.); 2 Department of Psychiatry, “Prof. Dr. Alexandru Obregia” Clinical Hospital of Psychiatry, 041914 Bucharest, Romania; 3 Department of Anatomy, Animal Physiology and Biophysics, Faculty of Biology, University of Bucharest, Splaiul Independenţei 91–95, 050095 Bucharest, Romania; zamfirache.florin@s.bio.unibuc.ro; 4 Department of Educational Sciences, Faculty of Educational Sciences, Social Sciences and Psychology, The National University of Science and Technology POLITEHNICA Bucharest, Pitești University Centre, Targul din Vale, 1, 110040 Pitesti, Romania

**Keywords:** major depressive disorder, treatment-resistant depression, repetitive transcranial magnetic stimulation, theta-burst stimulation, non-invasive therapy

## Abstract

Major depressive disorder (MDD) is one of the most prevalent mental illnesses and, despite the availability of multiple treatment options, remains difficult to treat for a substantial proportion of patients. Repetitive transcranial magnetic stimulation (rTMS) is an important therapeutic approach for depression, modulating brain activity through targeted magnetic pulses in a non-invasive manner. This review examined scientific evidence from clinical trials, large-scale analyses, and laboratory investigations regarding the effectiveness and safety of rTMS, as well as its role within contemporary therapeutic strategies for depression. In addition to conventional rTMS, the review addresses theta-burst stimulation (TBS), deep TMS, and various approaches aimed at enhancing treatment efficacy or accelerating clinical response, while also discussing the practical utility of different stimulation devices. rTMS applied to specific regions of the prefrontal cortex has demonstrated significant antidepressant effects, and intermittent theta-burst stimulation (iTBS) appears to achieve comparable outcomes within a shorter time frame. Research further indicates that rTMS is associated with neurobiological changes in brain connectivity, modulation of neurotransmitter systems, and the promotion of neuroplasticity. Across studies, rTMS is generally considered safe, with reported adverse effects being mild and transient. However, further research is needed to optimize stimulation protocols, clarify medium and long-term effects, individualize treatment approaches, and determine the durability of therapeutic benefits.

## 1. Introduction

Major depressive disorder is one of the most common mental illnesses with a significant global impact, both socially and economically. Although pharmacological treatments and psychotherapy are widely used, a significant percentage of individuals continue to experience persistent symptoms or recurrent episodes that are resistant to conventional interventions [1]. It is estimated that over 100 million people suffer from depressive disorders [2], and their prevalence has increased in recent decades [3], making major depression a critical concern in contemporary mental health care and medical research.

Repetitive transcranial magnetic stimulation (rTMS) is a non-invasive treatment modality that can alleviate depressive symptoms by stimulating specific brain regions and modulating neuroplasticity. This therapeutic approach has gained increasing interest in recent years, largely because of the challenges associated with treating depression using conventional methods [4]. This technique involves the application of focused magnetic pulses to specific areas of the brain, leading to alterations in neural circuit activity and adaptive changes in brain connectivity [5]. Technically, a TMS system consists of a high-voltage power supply, an energy-storing capacitor, a coil that generates a magnetic field when activated, and an electrical switch that controls the conversion of the electrical current into magnetic pulses [6]. From a bioengineering perspective, rTMS can be conceptualized as a parameter-driven neuromodulation system in which coil design, stimulation depth, pulse frequency, and targeting precision interact to determine both neurobiological and clinical outcomes.

The scientific basis of this technology lies in the principle of electromagnetic induction, first described nearly two centuries ago [6]. In 1980, Anthony Barker and colleagues at the University of Sheffield developed the first functional transcranial magnetic stimulation device, initially referred to as “electromagnetic stimulation” [7]. Unlike other treatments, such as electroconvulsive therapy, which rely on direct application of electric currents, TMS operates by generating magnetic fields at the scalp. This enables stimulation of the targeted brain regions in various disorders without the need to penetrate the skin, muscle, or bone [8]. Through the delivery of focused electromagnetic pulses, rTMS modulates neuronal activity, and accumulating evidence indicates promising therapeutic outcomes across a range of clinical conditions [9]. As a mode of operation, the rTMS coil is positioned on the scalp. It produces short magnetic pulses that induce electrical currents in neurons located under the surface of the skull. These induced currents lead to neuronal depolarization and alter cortical excitability. Beyond transient effects, rTMS can produce lasting changes in functional brain organization by promoting synaptic plasticity and supporting recovery processes following neuronal dysfunction or injury. Neuroimaging research has also demonstrated that stimulation of a specific cortical area can modulate activity in distant, structurally and functionally connected brain regions [10]. At the cellular level, TMS primarily acts on intracortical axons, particularly myelinated axon terminals, which are more readily excitable [11]. The size of the electromagnetic field and the orientation of the axon relative to it determine the effectiveness of depolarization.

**Figure 1:**
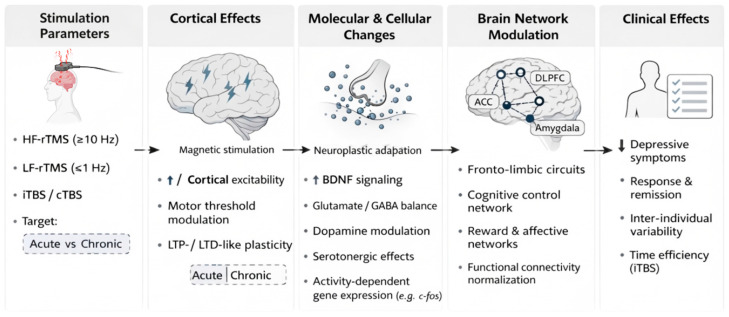
Multilevel mechanisms of rTMS in MDD. Variations in rTMS coil shape and parameters modulate synaptic and molecular plasticity by influencing local cortical excitability. The effects generate clinically significant antidepressant outcomes by propagation along fronto-limbic networks. Created in BioRender. Zamfirache, F. (2026) https://BioRender.com/v5lllq5 (accessed on 17 December 2025).

rTMS is an example of the translation of physics and engineering principles into clinically applicable medical solutions. Figure 1 illustrates the technical configuration and key procedural steps involved in rTMS, highlighting the link between device parameters and the resulting neurobiological and molecular effects. The efficacy and safety of rTMS in clinical practice depend on aspects such as coil shape, pulse type, intensity and frequency of stimulation, as well as the precision with which the target brain region is localized [11,12]. Dosimetry refers to the magnetic field characteristics set by the device. These include stimulation amplitude, width, frequency, waveform, configuration, and placement of equipment or devices [13]. These properties control the distribution and strength of the electromagnetic field, which is critical to the physiological response and treatment of the patient.

Computer modeling studies have shown that even minor variations in coil design or positioning can affect the extent of cortical stimulation and the intensity of the induced electric field. Such variations may account for interindividual differences in physiological and clinical responses to otherwise identical treatment protocols [12].

The coil type should be selected based on the targeted brain region and the intended clinical purpose. No coil perfectly combines magnetic field depth and focus. Instead, the design of electromagnetic coils determines the penetration depth of the electromagnetic field and the area of focus. Recent advances in device engineering, such as the development of figure-of-eight coils and H-coils, have enabled more focal and/or deeper stimulation of specific brain regions, thereby expanding the range of viable therapeutic targets [14]. According to Deng, Lisanby, and Peterchev [15], figure-of-eight coils generate shallow but more focused electric fields. Large circular coils penetrate tissues deeper than H-coils but are also less focused. Double cone coils can stimulate deeply and relatively focused, and the angle between their wings can increase the depth (up to ~110°), after which the effect reverses.

In addition to coil design, the effectiveness of TMS is influenced by the spatial distribution of the induced electric field. Modern computational modeling techniques can predict this distribution with high accuracy, enabling more precise and individualized targeting of cortical regions [16]. Also, the integration of rTMS with neuronavigation systems based on neuroimaging data and functional connectivity analyses has significantly increased the precision of stimulation and paved the way for brain network-centric approaches that allow protocols to be tailored to the neurobiological characteristics of each patient [17,18]. Also, combining rTMS with complementary neurophysiological and neuroimaging methods—such as electroencephalography (EEG) and functional magnetic resonance imaging (fMRI)—provides objective measures for monitoring stimulation-induced changes in brain activity and connectivity [19,20]. Overall, advances in neuromodulation techniques towards greater precision, along with the refinement of TMS protocols for treating depression, have been emphasized in bioengineering studies aiming to improve clinical outcomes.

While existing reviews typically focus on clinical efficacy or guideline-based recommendations, this review integrates evidence from clinical trials, large-scale meta-analyses, and laboratory-based studies to provide a comprehensive synthesis of rTMS effects in MDD. By examining antidepressant outcomes, safety profiles and underlying neurobiological mechanisms, such as alterations in brain connectivity, neurotransmitter modulation, and neuroplasticity, the review aims to provide a translational framework connecting clinical efficacy with biological processes. In addition to conventional rTMS, this scoping review systematically examines intermittent theta-burst stimulation (iTBS), deep TMS and accelerated or efficacy-enhancing stimulation paradigms.

## 2. Methodology

This study was conducted as a scoping review, following established methodological guidance for scoping studies. The aim of the review was to map the breadth of existing evidence, theoretical frameworks, and mechanistic insights related to repetitive transcranial magnetic stimulation (rTMS) in major depressive disorder, rather than to perform a formal systematic synthesis or meta-analysis. This approach was chosen over a quantitative meta-analysis because the study aimed to present the overall picture, particularly technological developments in the field, mechanistic insights, and clinical findings. Specifically, the objectives of this review were

O1. To investigate the literature on TMS interventions in depression.

O2. To analyze evidence regarding the mechanisms of action, efficacy, and safety of TMS.

O3. To summarize current trends and advances, highlighting insights that may inform future research and clinical practice.

### 2.1. Search Strategy

To ensure a synthesis of the current landscape of rTMS in MDD, a literature search was conducted in major scientific databases (e.g., PubMed/MEDLINE, Scopus, and Web of Science) to identify relevant peer-reviewed articles published in English. The search focused on peer-reviewed articles published between January 2000 and November 2025, using key terms such as “transcranial magnetic stimulation and depression, “theta burst stimulation and depression,” neuromodulation and depression and TMS” and “clinical translation and depression and TMS.” We selected all relevant studies and examined their reference lists to identify additional sources. Studies were considered based on their contribution to understanding the neurobiological mechanisms, clinical applications, and technological developments of TMS, with particular attention to randomized controlled trials (RCTs), open-label trials, systematic reviews, narrative reviews, and large-scale meta-analyses that bridge the gap between neurobiological theory and clinical practice.

Articles were excluded if they lacked primary data, focused on non-depressive psychiatric conditions, or were published in languages other than English. This structured approach was employed to ensure a comprehensive and representative mapping of the literature. The search strategy was intentionally broad to ensure inclusive coverage of the literature, consistent with the exploratory nature of a scoping review.

### 2.2. Inclusion and Exclusion Criteria

Studies involving human participants with depressive disorders receiving TMS and reporting outcomes related to depressive symptoms or the neurobiological, neurophysiological, or neuroimaging effects of TMS were included. Studies using advanced protocols, including theta-burst stimulation, deep TMS, and accelerated treatment approaches, were incorporated. Single case studies, editorials, conference abstracts, and studies lacking sufficient methodological details or relevant results were excluded from the analysis.

Included studies were selected based on their design, sample size, methods used to diagnose depression and reported outcomes. Attention was given to how TMS protocols were described to capture relevant methodological and clinical information. This approach aimed to provide a comprehensive overview of the current literature and reflect contemporary practice in the field.

In addition to the qualitative synthesis of clinical and mechanistic evidence, this review incorporates specific bibliometric analyses to provide a quantitative backdrop to the “bench-to-bedside” transition of rTMS. These metrics, including publication trends, core journals and institutional contributions, objectively demonstrate the exponential growth and expanding global interest in rTMS research over the last two decades.

### 2.3. Data Analysis

Records identified through database searches were screened in a stepwise manner based on titles and abstracts, followed by full-text review where appropriate. Inclusion criteria encompassed original research articles, clinical trials, observational studies, and relevant reviews addressing rTMS mechanisms or clinical applications in depression. Exclusion criteria included non-peer-reviewed sources, conference abstracts without full text, and studies not directly relevant to the scope of the review. Key information from included studies was charted descriptively, focusing on study characteristics, stimulation protocols, proposed mechanisms of action (neurophysiological, network-level, molecular), and clinical implications. Data were synthesized narratively to highlight patterns, converging evidence, and areas of divergence across the literature. No quantitative synthesis, meta-analysis, or formal assessment of study quality or risk of bias was undertaken, as these approaches fall outside the objectives of a scoping review.

## 3. Neurophysiological Effects of Transcranial Magnetic Stimulation

### 3.1. Acute Effects

The effects of TMS depend directly on the cortical area stimulated and the parameters employed. When applied over the primary motor cortex, TMS elicits measurable muscle responses, also referred to as motor evoked potentials (MEPs). These are used as indicators of excitability. The most common response is a contraction of the muscles on the side of the hand contralateral to the stimulated motor cortex area [21].

When TMS is applied over the occipital cortex, it can be associated with brief visual sensations, known as phosphenes, whereas stimulation of specific language or association areas may produce transient speech disruption or interfere with cognitive processing [22]. This evidence highlights how TMS modulates brain activity and can selectively disrupt cortical function in the stimulated regions.

Motor threshold measurement is one of the most widely used methods for assessing cortical responsiveness to TMS stimulation. It is defined as the lowest stimulation intensity required to produce a consistent motor evoked potential (MEP). Stimulation is typically adjusted so that a motor response occurs in at least half of the trials, serving as a standard measure of corticospinal excitability [10,21]. Various biological and experimental factors influence motor threshold values. These include genetic factors, hormonal levels, sleep deprivation, neurological disorders, and medications. Changes in motor threshold following TMS stimulation may indicate inhibitory effects when thresholds increase or facilitatory effects when thresholds are decreased [11].

The success of TMS treatment depends on the precise and consistent localization of the stimulation site. When targeting the motor cortex, the coil must be positioned 5 cm anterior to the vertex, along the parasagittal line [19]. For TMS treatment of depression, the prefrontal cortex is targeted, located approximately 5 cm anterior to the motor hotspot, corresponding to position F3 on the standard 10–20 EEG map [22]. Another important factor is the coil orientation: when tilted at 45 degrees to the sagittal plane, it produces the largest MEPs, indicating more efficient activation of cortical neurons and underlying nerve fibers [22].

The specific orientation of the coil has received less attention compared with the use of functional connectivity and network mapping to guide the TMS targeting in recent studies. In clinical practice, two main coil positioning approaches are commonly used: a parasagittal orientation directed towards the nasion and set at 45 degrees to the sagittal plane, or a second orientation chosen primarily for convenience rather than physiological reasons [23]. As emerging evidence suggests that coil orientation can influence the distribution of induced electric fields in the brain and the specific neural networks activated, further research is needed to optimize TMS outcomes for individual patients.

### 3.2. Chronic Effects

rTMS can modulate neuronal excitability depending on the frequency used in the stimulation protocol [22,24]. These changes are thought to result from synaptic modifications induced by stimulation and reflect core processes of brain plasticity, including long-term potentiation (LTP) and long-term depression (LTD), which are essential processes in learning that are often disrupted in depression [25].

Over time, these functional changes are consolidated, and repeated stimulation supports the restoration of synaptic circuits [6].

Continuous sessions of rTMS affect, at the cellular and molecular levels, signaling pathways crucial for neuroplasticity. This can lead to changes in the levels and activity of neurotrophic factors, especially brain-derived neurotrophic factor (BDNF), which is one of the essential factors in synaptic restructuring, neurogenesis, and mood regulation [22,26].

Clinically, the long-term effects of rTMS are mediated by its capacity to reconfigure brain networks, thereby normalizing neurotransmitter activity and promoting synaptic plasticity. Collectively, these observations highlight the need to adjust treatment protocols—such as the number of sessions, the length of time between sessions, and the stimulation frequency required for lasting effects and benefits for individuals with depression.

### 3.3. Side Effects and Safety Considerations

rTMS (transcranial magnetic stimulation) is generally safe and well-tolerated when established clinical safety protocols are followed. Reported adverse effects are usually mild and may include scalp discomfort at the stimulation site or headaches. Side effects are generally reported to be temporary, resolving spontaneously shortly after administration or, where appropriate, with analgesics. Although extremely rare, rTMS can trigger epileptic seizures, particularly in individuals with preexisting neurological risk factors or if established safety parameters are not properly followed [27].

Among individuals with psychiatric conditions, isolated cases have been reported in which rTMS—most commonly targeting the left prefrontal cortex—was associated with the onset of hypomania or mania in patients with mood disorders. However, systematic reviews and meta-analyses indicate that the rate of mood switching with rTMS does not exceed that typically observed in bipolar disorder and is not higher than with other antidepressant therapies [25]. Similarly, although there are occasional reports of new psychotic symptoms or suicidal thoughts occurring during rTMS sessions, the available evidence does not demonstrate a clear causal relationship between rTMS and these adverse psychiatric outcomes [28].

Apart from its effects on clinical symptoms, rTMS may modulate temporary changes in neuroendocrine function. Some studies have observed short-term shifts in hormone levels—particularly prolactin and thyroid-stimulating hormone—after rTMS sessions [29,30]. However, investigations into the effects of rTMS on the hypothalamic–pituitary axis have yielded mixed results, and to date, there is no strong evidence that rTMS causes lasting disruptions in endocrine function.

## 4. Clinical Evidence and Application of rTMS in Depression

As an FDA-approved neuromodulation technique, TMS is increasingly used in the clinical management of depressive disorders. The U.S. Food and Drug Administration (FDA) first approved TMS in 2008 for the treatment of MDD in patients who failed to respond to at least one antidepressant medication. In parallel, both the FDA and the UK National Institute for Health and Care Excellence (NICE) have recognized TMS as a therapeutic option for migraine [31]. The use of TMS in clinical practice has yielded increasing results for anxiety-related disorders, including post-traumatic stress disorder (PTSD), as well as substance use disorders [32,33]. Since 2009, the FDA has approved TMS for the treatment of obsessive–compulsive disorder (OCD), and research continues for neurological disorders such as epilepsy. In clinical practice, for the aforementioned disorders, including smoking cessation and depression, repeated magnetic pulses, known as rTMS, are administered [22].

A total of 5046 articles on the use of TMS in major depressive disorder were published between 1999 and 2023. A total of 804 journals have published articles on this topic, with Brain Stimulation publishing the most. Over 4573 institutions from 77 countries and 16,000 authors have contributed to research in this field, highlighting the growing scientific interest in neuromodulation. The main research interests include analyzing neural networks modulated by rTMS, precisely identifying target brain regions and evaluating safety and therapeutic efficacy.

The clinical results synthesized from multiple large-scale studies, such as meta-analyses and randomized controlled trials conducted on rTMS, reflect substantial heterogeneity. Differences between studies are reported in terms of response to treatment, variability in the number of sessions, stimulation parameters, coil position, and aspects related to patient characteristics. Furthermore, current remission rates are modest, indicating the need for further refinement of treatment protocols and implementation of individualized therapeutic strategies. Notably, placebo or sham responses are non-negligible, reaching rates of up to approximately 25% in some trials, which complicates the interpretation of efficacy outcomes. This reinforces the idea that studies with rigorous protocol controls and robust sample sizes are necessary (Table 1).

### 4.1. Protocols of Transcranial Magnetic Stimulation in Depression

TMS is a non-invasive neuromodulation technique that operates through distinct pulse delivery paradigms designed to probe and modulate cortical excitability and synaptic plasticity. The principal stimulation protocols include single-pulse TMS (sTMS), paired-pulse TMS (ppTMS), and rTMS [39]. While sTMS and ppTMS were initially developed as experimental tools to investigate cortical function, connectivity, and excitatory–inhibitory balance, rTMS has evolved into a clinically established therapeutic modality in psychiatric practice [9]. When paired pulses are delivered to spatially distinct cortical or peripheral targets, the technique is referred to as paired associative stimulation (PAS), which enables the investigation of spike-timing–dependent plasticity mechanisms.

rTMS protocols widely applied in clinical practice for the treatment of depression use high-frequency stimulation of the left DLPFC to increase cortical excitability and low-frequency stimulation of the right DLPFC, hypothesized to induce inhibitory effects [40].

Maximum antidepressant effects are achieved with high-frequency rTMS protocols delivering 1200–1500 pulses per session, as demonstrated by meta-analyses [9,41]. Reinforcing these conclusions, data from a large meta-analysis involving 2982 patients indicate that rTMS significantly reduces the severity of depressive symptoms while maintaining a high safety profile [38].

**Figure 2:**
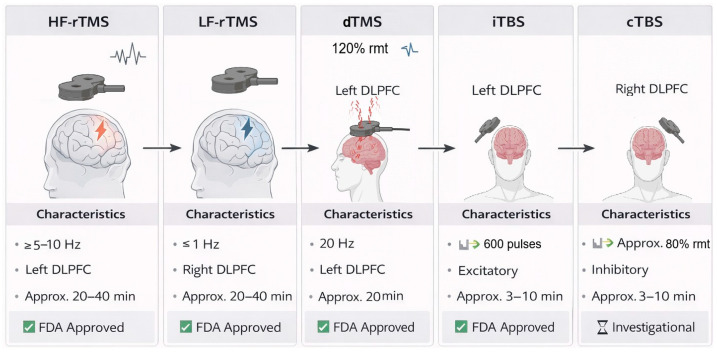
rTMS protocols and cortical effects. Created in BioRender. Zamfirache, F. (2026) https://BioRender.com/48iow70 (accessed on 17 December 2025).

Recent technological advances in TMS have enabled methods designed to enhance the spatial and temporal regulation of neuronal function (Figure 2). One example is TBS, an innovative protocol that mimics the natural endogenous theta rhythms of the hippocampus and promotes long-term synaptic potentiation [42]. The iTBS protocol, FDA-approved for treatment-resistant depression, offers the advantage of significantly shorter session durations while still eliciting robust neuroplastic effects compared to traditional rTMS [43,44]. In contrast, by producing inhibitory effects on cortical excitability, continuous theta-burst stimulation (cTBS) highlights the importance of adjusting stimulation polarity to optimize clinical outcomes [45]. An increasing number of techniques are being investigated to enhance efficacy in the treatment of depression, including prolonged iTBS (piTBS) and accelerated iTBS (aiTBS), which may exert cumulative neuroplastic effects and improve recovery [46]. A randomized controlled trial demonstrated the efficacy of both iTBS and conventional rTMS protocols in alleviating depressive symptoms, with iTBS showing superior short-term outcomes [26].

In parallel, advances in coil design have allowed the expansion of cortical areas targeted by rTMS. Deep rTMS (drTMS), delivered using the H-coil system, enables stimulation of deeper cortical and subcortical structures involved in mood regulation [47]. Clinical studies have demonstrated that high-frequency drTMS is both effective and well tolerated in patients with treatment-resistant major depressive disorder, with therapeutic benefits persisting for several months following treatment [14]. Collectively, these developments highlight how bioengineering-driven innovations in stimulation protocols and device design are progressively refining neuromodulatory strategies for depressive disorders.

### 4.2. Implications of Sham Effects

Substantial sham response rates, reported at approximately 20–25% in some trials, have important implications for both the design and the interpretation of rTMS study efficacy. The reported antidepressant effects suggest a combination of stimulation-specific neurobiological mechanisms and non-specific factors, such as patient expectancy regarding treatment outcomes. High sham responsiveness may indicate persistent limitations in binding, from a methodological perspective. Many sham stimulation protocols fail to accurately reproduce the sensory and auditory characteristics of active stimulation. From a bioengineering standpoint, these sham effects highlight the need to improve device design control conditions, as well as to incorporate objective physiological or neuroimaging biomarkers in future clinical trials, in order to reliably distinguish true neuromodulatory effects from placebo-related influences.

### 4.3. Brain Network Effects of rTMS in Depression

At the systems level, the therapeutic effects of rTMS are mediated by large-scale modulation of distributed brain circuits implicated in mood regulation, cognitive control, and stress responsivity. Accordingly, rTMS efficacy depends on the neuroanatomical and functional organization of the prefrontal cortex. In depression, stimulation is most commonly applied to the DLPFC, a key hub that is frequently disrupted in this disorder [48]. The prefrontal cortex consists of multiple subregions with distinct functional roles.

The vmPFC, located below the curvature of the corpus callosum and including the medial orbitofrontal region, is involved in emotional regulation, associative learning, and value-based decision-making via connections with limbic structures [49,50]. In contrast, the dorsolateral prefrontal cortex (DLPFC) supports higher-order cognitive processes, including working memory, attention, planning, and impulse control [48,51].

Although rTMS is applied focally to DLPFC, its clinical effects arise because this region serves as a central node within widely distributed functional networks. In major depressive disorder (MDD), rTMS modulates two networks strongly implicated in symptom expression and treatment response: the default mode network (DMN) and fronto-parietal network (FPN). The FPN, which includes the DLPFC and is involved in top-down attention, cognitive control, and working memory, whereas dmPFC, which contributes to DMN, regulates ruminative thoughts and emotional processing [52,53,54,55]. Within this framework, Kaiser et al. [56] documented significant alterations in resting-state functional connectivity in patients with MDD, such as hyperconnectivity within the default mode network and hypoconnectivity within the fronto-parietal network.

Consistent with this network-based perspective, although both stimulation targets attenuated depressive symptoms, they have been linked to distinct connectivity changes: DLPFC stimulation decreased negative connectivity with the precuneus, while dmPFC stimulation increased positive connectivity with the precuneus and posterior cingulate cortex (PCC) [55]. These network-level effects reflect underlying regional dysfunctions within the prefrontal cortex.

In major depressive disorder, prefrontal subregions exhibit imbalanced activity, hyperactivity in ventromedial prefrontal circuits, and hypoactivity in the dorsolateral prefrontal cortex [49]. Bradykinesia, along with decreased executive function and associated symptoms such as apathy, is attributed to hypofunction in these regions [27]. Thus, restoring functional balance necessitates addressing this asymmetry via neuromodulation.

Depression is influenced by alterations in the excitatory-inhibitory balance and impaired synaptic inhibition. GABAergic interneuronal circuits are implicated in mediating these effects. The use of rTMS protocols in target regions is recommended to enhance neuronal excitability or prevent maladaptive hyperactivity.

Neurophysiological studies in patients with depression have demonstrated reduced inhibitory control compared to healthy individuals. Decreased short-term neuronal inhibition (SICI) and long-term neuronal inhibition (LICI) have also been reported [57]. However, current evidence does not support the generalization that all patients with depression exhibit reduced short-term (SICI) or long-term (LICI) intracortical inhibition. Thus, in adolescents with suicidal ideation and treatment resistance, depressive disorders are associated with inhibitory deficits [58]. In contrast, adults with untreated or less severe major depressive disorder (MDD) exhibit normal inhibitory function [59].

Neuroimaging and neurophysiological studies further support this network-based view, demonstrating that rTMS effects propagate beyond the stimulation site through interconnected cortical and subcortical regions [60], including the anterior cingulate cortex, hippocampus, orbitofrontal cortex, amygdala, and cerebellum.

In major depressive disorder, the hippocampus exhibits significant functional and structural alterations and plays an important role in memory formation and emotion regulation [61]. Following rTMS, increased hippocampal activity has been observed, which was associated with a reduction in depressive symptom severity but showed no significant effects on anxiety or sleep disturbances. The orbitofrontal-hippocampal pathway may therefore mediate key aspects of the antidepressant response [62,63].

Electrophysiological evidence corroborates these imaging results. A group of 64 healthy subjects and 53 patients with major depressive disorder undergoing an EEG study showed diminished basal neural activity in the lateral prefrontal cortex, orbitofrontal cortex, and hippocampus. Following rTMS treatment, these regions exhibited a partial restoration of circuit dynamics along with a significant increase in activity [62].

Meta-analyses further indicate that depression is associated with functional and structural changes not only in the frontal regions but also in the brainstem and cerebellum [64].

Traditionally associated with motor control, the cerebellum has emerging evidence supporting its role in emotional and cognitive processing, due to its extensive connections with prefrontal and limbic areas. Accordingly, it may be considered a potential adjuvant target for rTMS in treating depression [65].

Supporting this association, positron emission tomography studies have reported increased baseline metabolic cerebral activity in patients with recurrent depression who underwent high-frequency rTMS protocols targeting the left DLPFC [64].

Another important node within depression-related circuits is the anterior cingulate cortex. rTMS protocols that enhance theta band activity in the rostral anterior cingulate cortex have been shown to promote clinical improvement following antidepressant treatment, and may serve as a potential predictive biomarker of treatment response [66,67].

Altered functional connectivity in depressive disorders has also been observed in the amygdala, a region involved in reward processing and stress reactivity.

**Figure 3:**
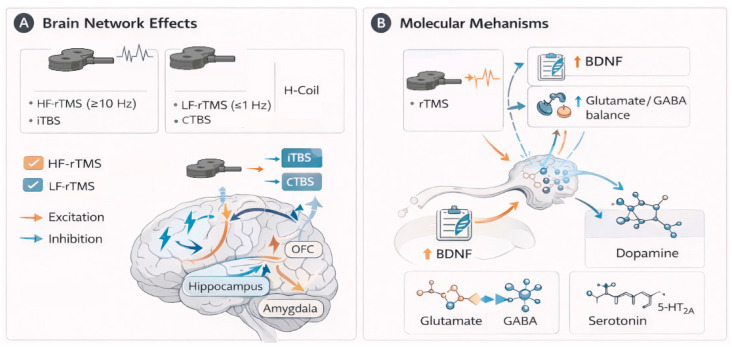
Brain network and molecular mechanisms of rTMS in depression. Solid orange arrows: Represent excitation. Solid blue arrows: Represent inhibition. Created in BioRender. Zamfirache, F. (2026) https://BioRender.com/uauu3ay (accessed on 15 December 2025).

Together, these results suggest that the antidepressant effects of rTMS are mediated by the modulation of distributed neural networks and that correcting these functional imbalances may benefit emotional regulation, stress resistance, and cognitive functions (see Figure 3).

### 4.4. Molecular Mechanisms of rTMS in Depression

At the molecular level, transcranial magnetic stimulation exerts antidepressant effects through activity-dependent neuroplastic mechanisms involving neurotrophic and gene-regulatory processes [68].

Converging evidence in humans supports this mechanism. rTMS may modulate changes in functional connectivity that overlap with regions expressing genes involved in synaptic plasticity and neural connectivity changes [69].

Preliminary evidence further indicates that rTMS can trigger activity-dependent gene expression changes. Hausmann [70] reported that high-frequency rTMS (20 Hz) increased expression of the immediate early gene c-fos across both superficial and deeper cortical layers, as well as within specific neuronal populations of the hippocampus. Evidence points to the possibility that rTMS engages intracellular signaling mechanisms that contribute to neuronal activation and synaptic changes. Nevertheless, the specific molecular pathway and long-term biological consequences of these effects remain incompletely understood and require further investigation.

Preclinical evidence provides insight into the intracellular processes underlying these effects. In rodent models, high-frequency rTMS has been shown to act as a neuroprotective agent, reducing neuronal apoptosis by regulating protein expression and optimizing cell metabolism [71]. Another recent study in rat models suggests that accelerated iTBS (aiTBS) can modulate the activity of specific subtypes of prefrontal neurons. Researchers observed recovered dendrites and neuronal spines during stimulation, mainly at the dmPFC level in the intratelencephalic neurons (IT) [56]. The results suggest that accelerated iTBS can induce synaptic plasticity in specific neurons, which may contribute to the relief of depressive symptoms caused by chronic stress. rTMS regulates the pathophysiological mechanisms of depression by involving several neurotransmitter systems such as serotonin, dopamine, GABA, and glutamate [72].

Animal studies suggest that rTMS can regulate both excitatory glutamatergic and inhibitory GABAergic neurotransmission [72]. In humans, magnetic resonance spectroscopy in the prefrontal cortex showed that high-frequency rTMS increases GABA concentrations, supporting a normalization of inhibitory mechanisms commonly dysregulated in depressive disorders [68].

Although several studies suggest that rTMS can modulate neurotrophic factors such as BDNF, evidence remains conflicting. A meta-analysis found that rTMS did not significantly increase serum BDNF, with effects varying according to stimulation parameters, frequency, treatment duration, and participant characteristics such as age and health status [68,73]. Moreover, specific external factors such as smoking may further modulate these effects, as high-frequency rTMS to the left DPLFC has been associated with unchanged effects or even reduced serum BDNF levels in some controlled studies [74].

Another essential molecular pathway is dopaminergic modulation. Dopamine release can be increased in areas involved in reward processing, particularly in the amygdala, striatum, and ventral tegmental area, due to the action of rTMS on the left prefrontal cortex, as indicated by positron emission tomography [75]. These changes improve depressive symptomatology, such as anhedonia and cognitive dysfunction. rTMS also modulates neurotransmitter systems, which likely contribute to both its therapeutic effects and certain side effects. However, its impact on dopamine appears weak or variable, depending on the stimulation protocol and the characteristics of the population studied. Some studies have reported inconsistent findings; for example, a PET study in patients with depression found no significant changes in striatal dopamine synthesis following chronic rTMS treatment [76].

Conversely, experimental studies have demonstrated that high-frequency stimulation to frontal brain regions can enhance dopaminergic activity. For instance, 20 Hz stimulation has been associated with increased dopamine levels in the hippocampus, and stimulation of the DLPFC has been shown to elevate dopamine release in the caudate nucleus [77]. These neurochemical effects may help explain the antidepressant and motivational benefits of rTMS, as well as rare side effects, such as mania, that can occur in susceptible individuals [28].

It should be noted that many proposed molecular and network-level mechanisms are based on indirect human evidence or translational findings, and should therefore be interpreted as hypotheses rather than established causal pathways.

In conclusion, even the serotonergic system was affected by the effects of rTMS, even though it was usually targeted by antidepressant pharmacotherapy studies have demonstrated serotonergic activity-modulating effects [20], as human studies have indicated that rTMS acts on central serotonergic receptor binding activities at peripheral serotonin sites [73]. In particular, the change in 5-HT_2_A receptor binding correlates positively with action on the dorsolateral prefrontal cortex and negatively with that of the hippocampus [78]. Thus, indicating the molecular changes specific to the relevant regions for treatment response.

## 5. Limitations

Despite the growing body of evidence supporting the TMS approach as a treatment for depression, several limitations constrain the interpretation and generalizability of the current findings. First, in meta-analyses and randomized controlled trials, the size of the effects is quite modest and extremely variable, with considerable heterogeneity between studies. This variability can be explained by major differences in stimulation parameters (intensity, coil type, frequency, target area and location, total pulse dose), as well as by the variability of patient populations, treatment refractoriness, disease severity, adherence to pharmacological treatments, and the presence of other psychiatric or somatic comorbidities. This diversity complicates the comparison of results between studies and may influence treatment efficacy. There are methodological inconsistencies in the studies, including outcome measurement, types of statistical analysis, and comparisons between them.

Second, contradictions between clinical results remain evident, as some well-designed studies have failed to demonstrate a clear superior effect of rTMS over sham stimulation. Furthermore, these differences highlight concerns regarding reproducibility and robustness, particularly in studies of small size or insufficient stimulation conditions. One of the major challenges of this method remains blindness, due to the auditory and somatosensory properties of TMS that make it difficult to create simulated procedures that allow reproducing the active intervention without cortical stimulation. As a result, the evaluation of the effectiveness can be complicated, due to the expectation and the placebo effect, both in the active group and in the control group.

A further significant limitation is the relative paucity of long-term follow-up data. Although the short-term antidepressant effect has been documented in many studies, there are a limited number of studies that have systematically evaluated the lasting effects of treatment response, relapse rates, or optimal ways to maintain remission. Standardized protocols are lacking in research environments, which limits reproducibility in clinical practice. Moreover, clinical studies are carried out in specialized academic centers that may not accurately reflect the reality of the wider patient population.

From a translational perspective, using rodent models for testing presents several challenges. Their brains are considerably smaller, exhibit different cortical folding, and have a distinct network organization. These anatomical differences result in markedly different electric field distributions compared with the human brain. An important aspect is that animal models do not accurately reproduce human depression. Behaviors such as immobility in the forced swim test provide only a partial simulation of symptoms seen in humans. The main reason is that cognitive symptoms and anhedonia are difficult to assess in animal models. In addition, the effects of the environment in which these animals live are often ignored. The fact that animal models do not reproduce complex cognitive and affective symptoms makes interpreting their behavior in the context of human depression difficult. This limits direct correlations between behavioral observations and depressive phenotypes in humans.

Unlike pharmacological approaches, rTMS cannot selectively target specific neuronal subtypes, synaptic populations, or molecular signaling pathways. Electromagnetic field stimulation does not have a precise target at the tissue level because it acts on an extensive surface of the cortex or subregions. The effects depend largely on dose, the cortical area stimulated, coil type and protocol. This may explain why its effects are often variable and indirect.

Overcoming these limitations will require uniform methodologies, large, multicenter studies, improved placebo practices, longer follow-up periods, and systematic analysis of predictors of treatment response to support individualization of treatment strategies.

## 6. Conclusions

Converging evidence from clinical trials, neuroimaging, and experimental research demonstrates that transcranial magnetic stimulation marks a considerable advance in the treatment of depressive disorder. At multiple levels of analysis, TMS effects on mood regulation have been reported through modulation of neurotransmitter systems, synaptic plasticity, and functional brain neural circuits. TMS contributes to a considerable improvement in depressive symptomatology in a significant proportion of patients by modulating the excitatory-inhibitory balance and facilitating adaptive neuroplasticity.

Notably, TMS is characterized by a favorable safety and tolerability profile. No significant side effects were reported, with those present being mild, self-limiting, and transient. Due to its non-invasive nature and the minimal risk of persistent or serious side effects, it is considered a valuable therapeutic method, especially for patients who do not respond or are not completely adherent to conventional pharmacological treatments. Current research studies the applicability of the method beyond depression in a wide range of psychiatric and neurological pathologies.

Considering translational aspects, it is necessary to refine the methodology and personalize the protocols for strengthening the TMS method. Stimulation target accuracy and therapeutic efficiency are expected through technological advances in the field of neuronavigation and multimodal integration with neuroimaging and electrophysiology. Simultaneously, attention to molecular mediators, such as neurotransmitter dynamics and brain-derived neurotrophic factors, can contribute to the detection of response biomarkers and can guide the individualization of treatments.

Overall, although significant challenges persist, the cumulative evidence supports TMS as an encouraging and clinically valuable intervention for depression. Continued rigorous research, technological innovation, and precision-oriented approaches will be essential to fully define its optimal role in psychiatric practice and to maximize its therapeutic potential.

## Figures and Tables

**Table 1:** Summary of major meta-analyses and large RCTs evaluating rTMS for depression, including sample size, stimulation protocol, outcome measures, effect sizes, response and remission rates, and notes on heterogeneity, variability, and sham/placebo effects.

Study/Meta-Analysis	Sample Size	Protocol	Outcome Measure	Effect Size/Response	Remission Rate	Notes/Placebo Considerations
O’Reardon et al. [34]	301	10 Hz left DLPFC, 20 sessions	MADRS	d = 0.55 vs. sham	14%	Sham response 5–10%, moderate effect
Berlim et al. [35]	1545	HF-rTMS left DLPFC	HAMD	Hedges’ g = 0.33	NR	Significant heterogeneity (I^2^ > 50%)
Li et al. [36]	2200	rTMS, various protocols	HDRS	SMD = 0.30	18%	Placebo response rates up to 25% in some trials
Chen et al. [26]	120	iTBS vs. 10 Hz rTMS	HDRS	iTBS: d = 0.65; 10 Hz: d = 0.21	iTBS: 28%; 10 Hz: 10%	Short-term follow-up, variability in patient characteristics
Lefaucheur et al. [37]	1000+	HF vs. LF rTMS	MADRS/HDRS	Response: 29–45%	15–30%	Strong site-dependent variability, sham effects noted
Dalhuisen et al. [38]	2982	rTMS	HAMD	Mean reduction 3.2 points vs. sham	22%	Significant variation in effect sizes, modest clinical impact

## Data Availability

Data are contained within the article.

## Abbreviations

The following abbreviations are used in this manuscript:

ACC	Anterior cingulate cortex
aiTBS	Accelerated intermittent theta-burst stimulation
AMPA	α-Amino-3-hydroxy-5-methyl-4-isoxazolepropionic acid (receptor)
BDNF	Brain-derived neurotrophic factor
cTBS	Continuous theta-burst stimulation
PCC	Posterior cingulate cortex
DBS	Deep brain stimulation
DLPFC	Dorsolateral prefrontal cortex
dmPFC	Dorsomedial prefrontal cortex
DMN	Default mode network
EEG	Electroencephalography
FPN	Fronto-parietal network
fMRI	Functional magnetic resonance imaging
GBD	Global Burden of Disease
GABA	Gamma-aminobutyric acid
HF-rTMS	High-frequency repetitive transcranial magnetic stimulation
iTBS	Intermittent theta-burst stimulation
IT	Intratelencephalic neurons
LICI	Long-interval cortical inhibition
LF-rTMS	Low-frequency repetitive transcranial magnetic stimulation
MDD	Major depressive disorder
MEP	Motor-evoked potential
MRI	Magnetic resonance imaging
PAS	Paired associative stimulation
PET	Positron emission tomography
RCT	Randomized controlled trial
rTMS	Repetitive transcranial magnetic stimulation
sgACC	Subgenual anterior cingulate cortex
SICI	Short-interval cortical inhibition
SPECT	Single-photon emission computed tomography
TBS	Theta-burst stimulation
tACS	Transcranial alternating current stimulation
tDCS	Transcranial direct current stimulation
TMS	Transcranial magnetic stimulation
TRD	Treatment-resistant depression
vmPFC	Ventromedial prefrontal cortex
